# GhLTPG1, a cotton GPI-anchored lipid transfer protein, regulates the transport of phosphatidylinositol monophosphates and cotton fiber elongation

**DOI:** 10.1038/srep26829

**Published:** 2016-06-17

**Authors:** Ting Deng, Hongyan Yao, Jin Wang, Jun Wang, Hongwei Xue, Kaijing Zuo

**Affiliations:** 1 Plant Biotechnology Research Center, School of Agriculture and Biology, Shanghai Jiao Tong University, Shanghai 200240, China; 2 National Key Laboratory of Plant Molecular Genetics, Shanghai Institute of Plant Physiology & Ecology, Chinese Academy of Sciences, 300 Fenglin Road, 200032 Shanghai, China; 3 Biotechnology Research Institute, Chinese Academy of Agricultural Sciences, Beijing, 100081, China

## Abstract

The cotton fibers are seed trichomes that elongate from the ovule epidermis. Polar lipids are required for the quick enlargement of cell membrane and fiber cell growth, however, how lipids are transported from the ovules into the developing fibers remains less known. Here, we reported the functional characterization of GhLTPG1, a GPI-anchored lipid transport protein, during cotton fiber elongation. *GhLTPG1* was abundantly expressed in elongating cotton fibers and outer integument of the ovules, and GhLTPG1 protein was located on cell membrane. Biochemical analysis showed that GhLTPG1 specifically bound to phosphatidylinositol mono-phosphates (PtdIns3P, PtdIns4P and PtdIns5P) *in vitro* and transported PtdInsPs from the synthesis places to the plasma membranes *in vivo*. Expression of *GhLTPG1* in Arabidopsis caused an increased number of trichomes, and fibers in *GhLTPG1-*knockdown cotton plants exhibited significantly reduced length, decreased polar lipid content, and repression of fiber elongation-related genes expression. These results suggested that GhLTPG1 protein regulates the cotton fiber elongation through mediating the transport of phosphatidylinositol monophosphates.

Cotton fibers are trichomes that initiate from the ovular epidermal cells^1^. The developmental process of cotton fibers consists of four overlapping growth stages including fiber initiation, fiber elongation, secondary wall deposition, and fiber maturation^2^. Once fiber development initiates, the fiber cells quickly enter into the elongation stage, which is characterized by rapid synthesis of the primary cell wall and accumulation of cellular constituents^3^. The cotton fibers can attain lengths of up to 3.0 cm after ~20 days’ elongation and mature fibers consist of 95% cellulose, 1.8% protein, and 0.3% fatty acids, whereas elongating cotton fibers at 10 days post anthesis (DPA) only contain 23% cellulose, 22% protein, and 5% fatty acids^4^,^5^.

During fiber elongation, continuous synthesis and transport of lipids and proteins are crucial for supporting the vigorous enlargement of vacuoles and plasma membrane^6^,^7^. Studies showed that fatty acids are deposited in several concentric cell-wall layers alternating with cellulose^4^, and the developing fiber cells can incorporate the majority of ^14^C-labeled UDP-glucose with a variety of polar lipids into the cell wall from 3 to 20 DPA^2^. Lipidomic analysis using fiber cells revealed that the contents of polar lipids, including phosphatidic acid (PA), phosphatidylcholine (PC), phosphatidylethanolamine (PE), phosphatidylinositol (PI), and phosphatidylglycerol (PG), are increased and reach the maximum level during fiber elongation from 5 to 14 DPA, and decrease thereafter^6^,^7^, indicating that phospholipids are required for fiber cell expansion. In addition, comparative transcriptomic analysis revealed the significantly up-regulated fatty acid biosynthesis during cotton fiber elongation^8^,^9^,^10^,^11^ and lipid metabolic processes are substantially reprogrammed in a ligon-lintless mutant^10^. Very long chain fatty acids (VLCFAs) promote the sphingolipid biosynthesis by up-regulating serine palmitoyltransferase^10^ and supplement of VLCFAs in the ovule culture medium significantly enhances the elongation rate of fiber cells.

In plants, long chain fatty acids, as the building blocks of membrane lipids, are incorporated into glycerolipids in the plastid through different metabolic pathways^12^,^13^. The VLCFAs are likely to be converted into phospholipids or sphingolipids that serve as precursors of cell components or modulate the expressions of target genes^11^. However, as newly-initiated fiber cells do not possess the ability to synthesize a large amount of phospholipids for rapid cell expansion, how phospholipids are transferred from the epidermal cells to the developing fiber cells during fiber elongation is still unknown. Identification of factors/proteins transporting lipids from the ovules into the developing fibers will help to illustrate the relevant mechanisms.

Previous studies suggested that lipid transfer proteins (LTPs) are likely the candidates delivering the phospholipids to developing cells^6^,^14^,^15^ considering that most LTPs are abundantly expressed in the epidermis of developing tissues^16^,^17^,^18^,^19^. LTPs contain a hydrophobic pocket capable of binding long chain fatty acids^17^,^20^, and studies showed that plant LTPs are involved in transporting lipids from the endoplasmic reticulum (ER) to plasma membrane (PM) and subsequently from PM to the cell exterior^21^,^22^,^23^.

GPI-anchored lipid transfer protein (LTPG) belongs to the G-type LTP family and is composed of an N-terminal signal peptide (SP) for secretion, an LTP domain, and a GPI domain for protein processing and membrane anchoring^24^. In plants, LTPGs participate in callose deposition and metabolism, as well as cuticle biosynthesis in the epidermis of inflorescence stems^19^,^25^,^26^. *Arabidopsis* seedlings with decreased *AtLTPG1* expression show a reduced wax load on the stem surface, indicating that LTPG is involved in cuticle deposition^18^,^19^,^26^. In cotton, several LTPs are highly expressed at the early stage of fiber development, while they are down-regulated in the fiberless mutant Xu142*fl*, particularly at the fiber elongation stage^8^,^9^,^10^,^27^,^28^, indicating the importance of LTPs in fiber elongation, however, whether these LTPs deliver polar lipids to the elongating fiber cells and the roles of them remains to be determined.

Although many studies have focused on the functional identification of genes/proteins involving in activation and repression of cotton fiber elongation^11^,^29^,^30^,^31^, the mechanisms underlying phospholipid transportation and their effects have been scarcely investigated. We here report the identification and functional characterization of a cotton gene *GhLTPG1* that is highly expressed in the developing fiber. Suppressed *GhLTPG1* resulted in the shorter fibers, which was attributable to the decreased lipids content in elongating fibers.

## Results

### *GhLTPG1* is highly expressed in elongating fibers and outer integument of cotton ovules

To identify the transporters that deliver polar lipids to the cell surface of the developing fiber, the transcriptome of developing cotton fiber was searched and a fiber-predominantly expressed (from 3 to 10 DPA) *LTPG* gene^11^,^27^^,^^32^,^33^, *GhLTPG1* (KR072649), was characterized. *GhLTPG1* encodes a typical GPI-anchored LTP containing a 22-aa signal peptide at N terminus, a lipid transfer domain (32–114 aa), and a potential GPI anchor site (165–196 aa) (Fig. S1a). Alignment of GhLTPG1 with the deduced cuticle transporters AtLPTGs showed the high similarity in LTP domain (Fig. S1b).

**Figure 1:**
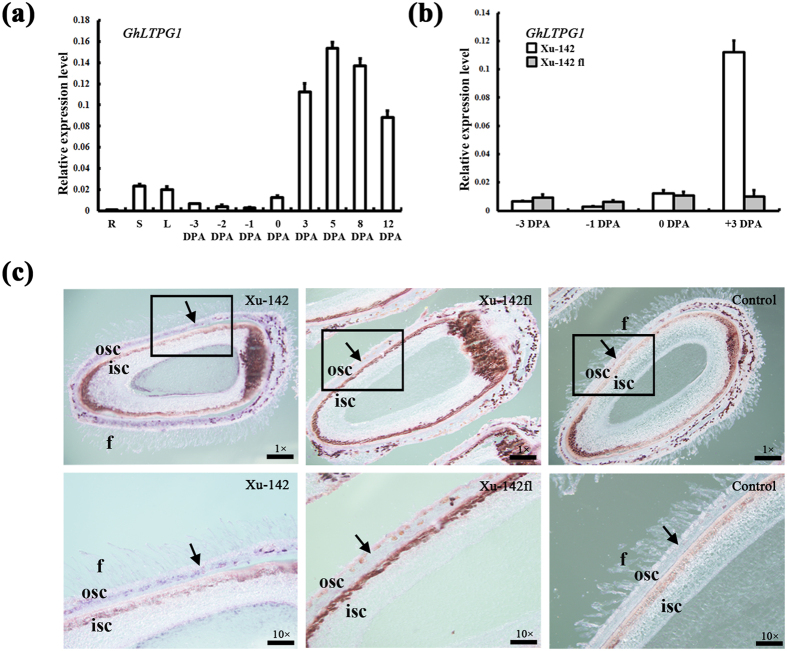
*GhLTPG1* is expressed in elongating cotton fibers and outer integument of cotton ovules. (**a**) *GhLTPG1* highly expresses in the ovules since cotton fibers initiate at 0 DPA, and lowly expresses in the ovules before 0 DPA (DPA, days post anthesis), roots (R), stems (S) and leaves (L). (**b**) *GhLTPG1* gene expression significantly increases in the ovules of Xu142 when fiber elongates (3 DPA), and no obvious expression change occurred in the ovules of Xu142*fl* (Xu142 fiberless and lintless mutant). (**c**) *GhLTPG1* gene mainly expresses in the fiber cells and outer integument of cotton ovule. *GhLTPG1* antisense probe detected *GhLTPG1* gene expression specific in the fibers and outer integument of Xu142 ovule at 3 DPA. Arrowhead indicates *GhLTPG1* expression signal (deep blue). No hybridization signal was observed in the Xu142*fl* ovule compared with Xu142 (arrowhead indicates no hybridization signal in osc). The sense probe displayed no signal in the ovule of Xu142, as the negative control. The bottom pictures are 10 times magnified images of corresponding upper panels respectively. f, fiber; isc, inner seed coat; osc, outer integument of seed coat. Scale bars: 200 μm in upper images (**c**); 100 μm in lower images (**c**).

Analysis by qRT-PCR revealed that *GhLTPG1* was lowly expressed in roots, stems, and leaves, while highly expressed in ovules after anthesis (Fig. 1a), suggesting a possible role of GhLTPG1 in fiber initiation/elongation. In addition, analysis of the spatiotemporal expression of *GhLTPG1* by expressing the *GhLPTG1* promoter driven GUS in *Arabidopsis* revealed the high expression of *GhLTPG1* in the vascular bundles of roots, the trichomes in different tissues including leaves, petals, and the stigma (Fig. S2).

Further comparative analysis of the *GhLTPG1* expression between Xu142 and corresponding fiberless mutant (Xu142*fl*) showed the increased expression after anthesis in Xu142 (reaches to 11-fold at 3 DPA), while no changes in Xu142*fl* (Fig. 1a,b), suggesting a close association of *GhLTPG1* transcription with fiber elongation. In addition, RNA *in situ* hybridization analysis using developing ovules confirmed the *GhLTPG1* expression in the developing fibers, outer seed-coat and embryo of Xu142 (Fig. 1c), while faint detectable in the corresponding areas of Xu142*fl* (Fig. 1c), further indicating the role of GhLTPG1 in fiber development.

### GhLTPG1 localizes on cell membrane requiring the signal peptide and GPI domain

**Figure 2:**
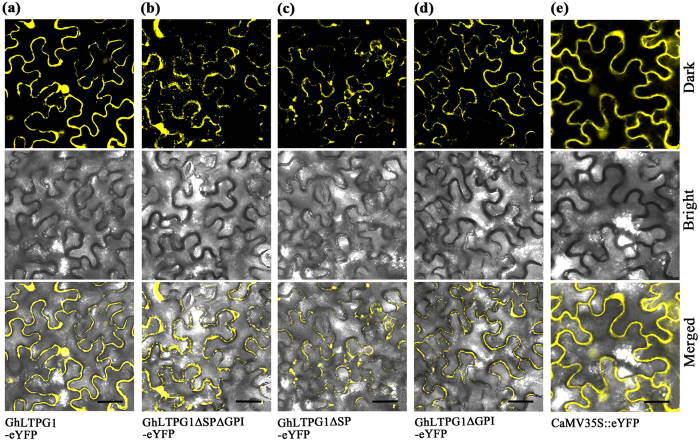
GhLTPG1 is localized in plasma membrane and nuclear membrane, and deletion of signal peptide or GPI domain or both results in uneven localization in plasma membrane. (**a**) GhLTPG1 without signal peptide and GPI domain fused with eYFP. (**b**) GhLTPG1 without signal peptide fused with eYFP. (**c**) GhLTPG1 without GPI domain fused with. (**d**) GhLTPG1 fused with eYFP. (**e**) Tobacco epidermis cells transformed with CaMV35S::eYFP was used as the control. Upper column: fluorescent image in dark field. Middle column: bright field. Lower column: the overlay of fluorescent signals and bright field images. Scale bar: 50 μm.

GhLTPG1 and various variants deleting different domains (GhLTPG1ΔSP, GhLTPG1ΔGPI, GhLTPG1ΔSPΔGPI) were fused with eYFP and transiently expressed in tobacco leaves to analyze the subcellular localization of GhLTPG1. The observation of GhLTPG1 subcellular localization showed that the fluorescent signals of GhLTPG1-eYFP were detected at the cell membrane (Fig. 2a, Fig. S3). GhLTPG1 deleting SP domain (GhLTPG1ΔSP, GhLTPG1ΔSPΔGPI) was accumulated in the cytoplasm and unevenly in the plasma membrane (Fig. 2b,c), indicating that the signal peptide is required for the plasma membrane targeting of GhLTPG1. GhLTPG1 deleting GPI domain (GhLTPG1ΔGPI, GhLTPG1ΔSPΔGPI) was unevenly distributed at the plasma membrane (Fig. 2b,d), indicating that the GPI domain is also associated with the GhLTPG1 distribution in plasma membrane.

Considering specific expression of *GhLTPG1* gene in cotton fiber and outer integument of ovule, we further analyzed protein localization of GhLTPG1 in different tissues. Interestingly, observation of the fluorescence showed that GhLTPG1-eYFP was located at the epidermis cells of leaves, trichome surface and outer integument of seed-coat (Fig. S4), while the truncated GhLTPG1 proteins (GhLTPG1ΔSP, GhLTPG1ΔGPI, GhLTPG1ΔSPΔGPI) were located at the surface of leaf trichomes and leaf epidermal cells (Fig. S4). As to the leaf cells, the effects of domain deletions on protein subcellular localization were similar to above results in tobacco system. The truncated GhLTPG1 proteins without SP domain (GhLTPG1ΔSP, GhLTPG1ΔSPΔGPI) expressed weakly in the epidermal cell of leaves. The signals of GhLTPG1 proteins without GPI domain (GhLTPG1ΔGPI, GhLTPG1ΔSPΔGPI) were not smoothly distributed as GhLTPG1 in plasma membrane of leaf epidermal cells. In summary, GhLTPG1 protein is targeted to cell membrane at subcellular level and has tissue-specific distribution.

### GhLTPG1 specifically binds and transports phospholipids PtdInsPs *in vitro*

**Figure 3:**
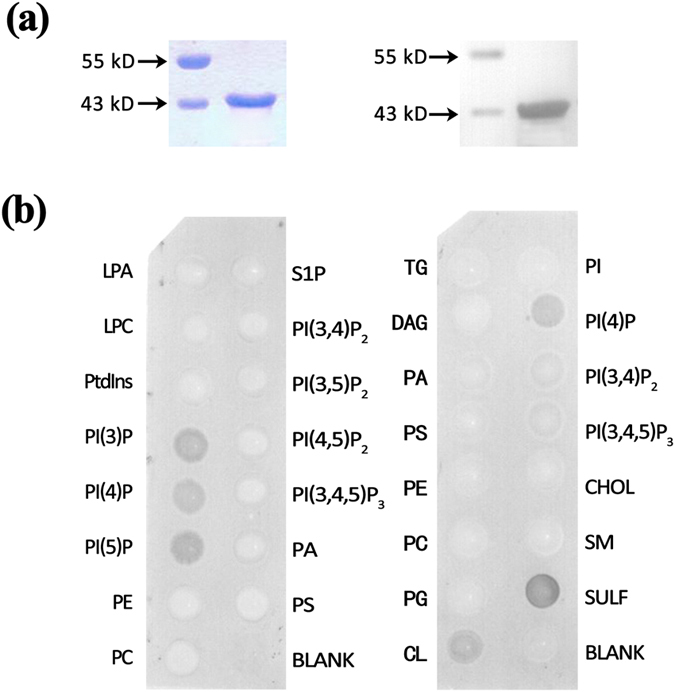
GhLTPG1 protein binds to phosphatidylinositol monophosphates (PtdInsPs). (**a**) SDS-PAGE separation (left) and immuno-blotting (right) of GhLTPG1 (only LTP domain)-GST. (**b**) GhLTPG1 specifically binds to phosphatidylinositol monophosphates (PtdInsPs) including PI(3)P, PI(4)P and PI(5)P (left), and binds to SULF (3-sulfogalactosylceramide) and CL (Cardiolipin) (right). LPA, Lysophosphatidic acid; LPC, Lysophosphocholine; PI(3)P, Phosphatidylinositol(3)-phosphate; PI(4)P, Phosphatidylinositol(4)-phosphate; PI(5)P, Phosphatidylinositol (5)-phosphate; PE, Phosphatidylethanolamine; PC, Phosphatidylcholine; S1P, Sphingosine 1-Phosphate; PI(3,4)P2, Phosphatidylinositol(3,4)-bisphosphate; PI(3,5)P2, Phosphatidylinositol(3,5)-bisphosphate; PI(4,5)P2, Phosphatidylinositol(4,5)-bisphosphate; PI(3,4,5)P3, Phosphatidylinositol (3,4,5)-trisphosphate; PA, Phosphatidic acid ; PS, Phosphatidylserine; TG, Triglyceride; DAG, Diacylglycerol; PG, Phosphatidylglycerol; PI, Phosphatidylinositol; CHOL, Cholesterol; SM, Sphingomyelin; BLANK, negative control. Different types of PIs: PI(3)P, PI(4)P, PI(5)P, PI(3,4)P2, PI(3,5)P2, PI(4,5)P2, and PI(3,4,5)P3.

To test the lipid binding activity of GhLTPG1, the LTP domain of GhLTPG1 was fused with a GST-tag and expressed for the protein-lipid binding assay *in vitro*. The recombinant protein was purified (~43 kDa, Fig. 3a) and analysis showed that GhLTPG1 (LTP domain)-GST strongly bound to the phosphatidylinositol monophosphates (PIs: PtdIns3P, PtdIns4P, PtdIns5P), 3-sulfogalactosylceramide and cardiolipin (Fig. 3b) among the tested phospholipids (PA, PC, PG, PE, PS, and PI).

**Figure 4:**
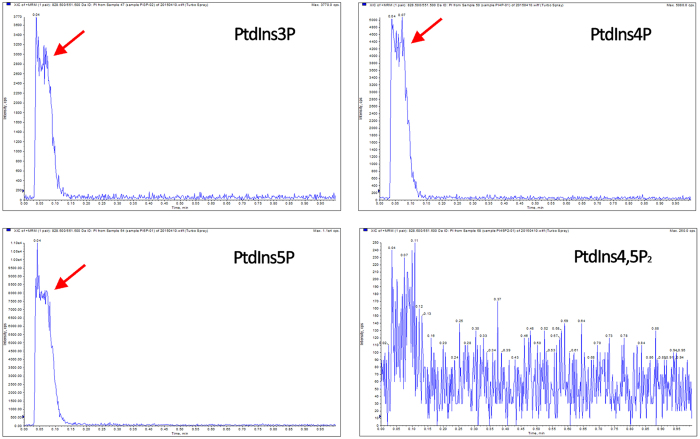
GhLTPG1 protein specifically transfers PtdInsPs *in vitro*. GhLTPG1 specifically transfers PtdIns3P, PtdIns4P, and PtdIns5P from heavy donor liposomes to light acceptor liposomes, but cannot transfer PtdIns(4,5)P_2_. Arrows indicate the peak of analyzed phosphatidylinositol in acceptor liposomes using LC-MS/MS.

Further, the lipid transfer activity assay was performed using liposomes *in vitro*. Donor liposomes prepared with PC/16 C-PtdIns3P, PC/16 C-PtdIns4P, PC/16 C-PtdIns5P, or PC/16 C-PtdIns(4,5)P_2_, respectively, were incubated with GhLTPG1 protein and acceptor liposomes (PC only), and the transfer activity was determined by mass spectrometry to examine whether PtdIns3P, PtdIns4P, PtdIns5P, or PtdIns(4,5)P_2_ was transferred into the acceptor liposomes. Due to the instability of phosphatidylinositol phosphates to be cleaved into phosphatidylinositol (PI), the existence of PI in acceptor liposomes was analyzed. Results showed that strong intensity of PI could be detected in all extractions of acceptor membranes for transferring PtdIns3P, PtdIns4P and PtdIns5P, respectively, but not PtdIns(4,5)P_2_ (Fig. 4). These results suggested that GhLTPG1 can specifically transfer the phosphatidylinositol monophosphates *in vitro*.

### GhLTPG1 transfers phospholipids to plasma membrane *in vivo*

**Figure 5:**
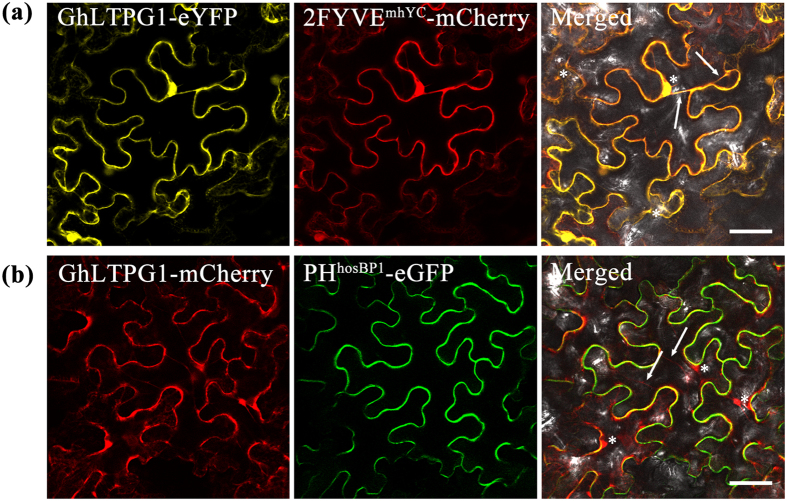
GhLTPG1 colocalizes with the biomarkers of PtdIns3P (2xFYVE^mHrs^-mCherry) and PtdIns4P (PH^hOSBP1^-eGFP) in cell membrane of tobacco epidermis cells. (**a**) GhLTPG1 colocalizes with PtdIns3P in cell membrane of tobacco epidermis cells. Subcellular localization of GhLTPG1-eYFP (left) and PtdIns3P (2xFYVE^mHrs^-mCherry) in tobacco epidermis cells (middle) was observed. The overlay of GhLTPG1 and PtdIns3P is shown (right). Bars = 50 μm. (**b**) GhLTPG1 colocalizes with PtdIns4P in cell membrane of tobacco epidermis cells. Subcellular localization of GhLTPG1-mCherry (left) and PtdIns4P (PH^hOSBP1^-eGFP) in tobacco epidermis cells (middle) was observed. The overlay of GhLTPG1 and PtdIns4P marker is shown (right). Bars = 50 μm. Stars and arrows indicate the fluorescently labeled GhLTPG1 formed into vesicle and moved along the filament, respectively.

The transfer activity of GhLTPG1 *in vivo* was further examined by using the marker lines, 2xFYVE^mHrs^-mCherry and PH^hOSBP1^-EGFP, to monitor the localization and dynamic changes of PtdIns3P and PtdIns4P, respectively^34^,^35^. Observations showed that both 2xFYVE^mHrs^-mCherry and PH^hOSBP1^-EGFP colocalized with GhLTPG1 at the plasma membrane of tobacco epidermal cells (Fig. 5a,b).

Further, the dynamic properties of PtdIns3P or PtdIns4P and GhLTPG1-eYFP/eRFP were tracked by continuous photographing. Results showed that PtdIns4P and GhLTPG1 interaction had a low association at membrane (video not shown). In contrast, the GhLTPG1-PtdIns3P complex in the cytoplasm moved along the filaments in two different ways (Video S1). The majority of GhLTPG1-PtdIns3P were loaded in small vesicles and fused to the cell membrane and a minority of the particles was likely targeted to the nuclear membrane. These results demonstrated that GhLTPG1 transports PtdInsP molecules to plasma membrane.

### *GhLTPG1* suppression retards the fiber elongation and affects the lipid profiles in cotton fiber

**Figure 6:**
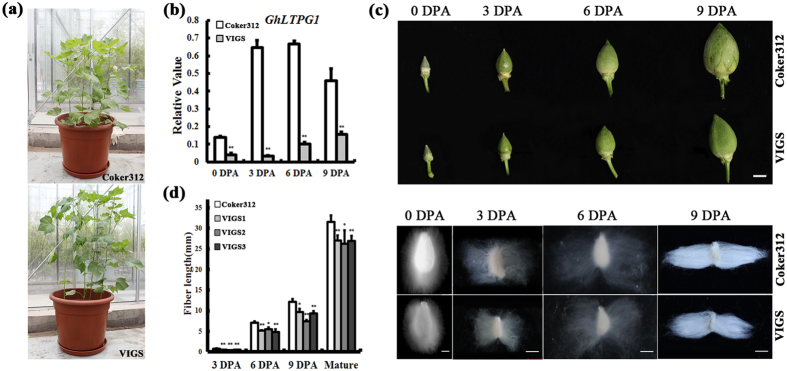
Suppressed *GhLTPG1* gene expression results in shorter cotton fibers. (**a**) No vegetative growth difference was observed between Coker312 and *GhLTPG1* gene VIGS cotton plants (VIGS, virus induced gene silencing). (**b**) *GhLTPG1* gene expression is knocked down in the fibers of *GhLTPG1* gene VIGS plants by qRT-PCR (**, P < 0.01). (**c**) The bolls of *GhLTPG1* VIGS cotton plants are smaller than those in Coker312. Bars = 1 cm. (**d**) Fiber length of *GhLTPG1* gene VIGS cotton plants at different stages is shorter than that of Coker312 (left), VIGS 1, 2, and 3 are three independent *GhLTPG1* gene VIGS lines; Suppressed *GhLTPG1* gene expression results in shorter cotton fibers (right) (*, P < 0.05; **, P < 0.01). Upper and lower panels show the fibers of Coker312 and *GhLTPG1* VIGS cotton plants respectively at 3, 6, 9 DPA and mature stage. Bars = 1mm (3 DPA), 5 mm (6–9 DPA); 1 cm (mature fibers).

To explore the physiological roles of *GhLTPG1* during fiber elongation, 20 independent VIGS cotton plants (variety Coker312) were generated using a *GhLTPG1* gene-specific fragment as the VIGS target. qRT-PCR analysis showed that the *GhLTPG1* expression was significantly reduced in the VIGS plants, although the vegetative growth of VIGS plants was not affected (Fig. 6a,b). The balls were smaller than those of wild-type (Fig. 6c), and all the *GhLTPG1* RNAi plants exhibited shorter fibers, which was confirmed by the detailed measurement of fiber lengths (Fig. 6d). At 3 DPA, fiber cells in wild-type plants reach up to 500 μm in length, whereas those of VIGS plants are only 150–200 μm long (Fig. 6d).

**Figure 7:**
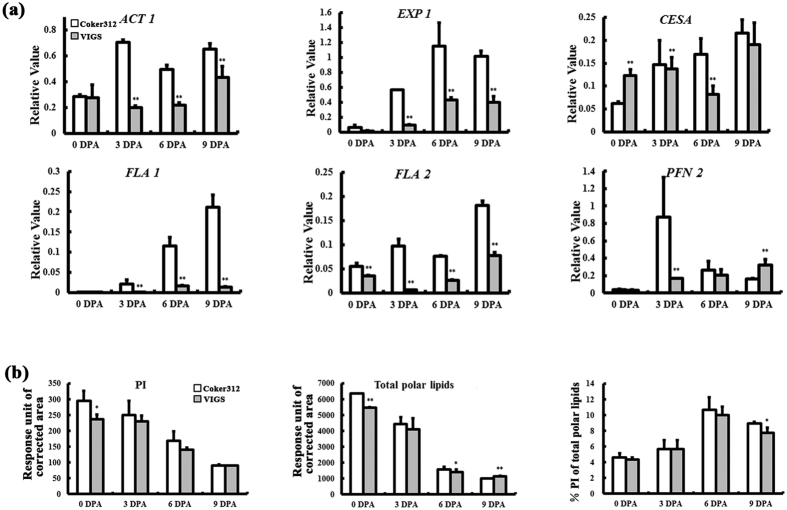
Suppressed *GhLTPG1* gene expression results in lower expression of fiber elongation-associated genes and lower polar lipid in cotton fiber. (**a**) The expression levels of six fiber elongation related genes in Coker312 are lower than those of VIGS plant at different stages (0, 3, 6, 9 DPA) (**, P < 0.01). (**b**) The ovules of *GhLTPG1* VIGS plants have lower amount of PI (phosphatidylinositol) and total polar lipids than those of Coker312, and the percentage of PI/total polar lipids in *GhLTPG1* silenced ovules is reduced. Asterisks denote the values significantly different from the corresponding controls (*, P < 0.05; **, P < 0.01) by t-test analysis.

Whether down-regulation of *GhLTPG1* affects the expression of fiber-elongation related genes was examined. Analysis using developing fibers showed that expressions of *ACTIN1, EXPANSIN 1* and *CESA* genes that directly participate in cell expansion and cellulose synthesis during fiber elongation were significantly reduced in VIGS plants (Fig. 7a), which is consistent with the shorter fibers of *GhLTPG1* VIGS plants.

Further, the polar lipids of fiber cells at early stage of elongation (0–6 DPA) were analyzed by HPLC-MS to check whether the shorter fibers in the VIGS plants resulted from a reduction of the lipid content. Results showed that the total polar lipid content continuously decreased during fiber development from 0 to 9 DPA in both wild type and VIGS plants (Fig. 7b). As to the components of polar lipids, the contents of PA, PE, PG and PS (phosphatidylserine) in the ovules were slowly decreased during fiber elongation and had no significant difference between wild type and VIGS plants. Conversely, the PI content significantly decreased in VIGS plants, and the percentage of PI/total polar lipid were 10–15% less in VIGS plants during all the examined stages (0 to 9 DPA) (Fig. 7b). Overall, *GhLTPG1* suppression resulted in the PI reduction, which was consistent with the shorten fiber length and reduced expressions of fiber elongation related genes.

### Ectopic expression of *GhLTPG1* enhances the leaf trichome number of *Arabidopsis*

**Figure 8:**
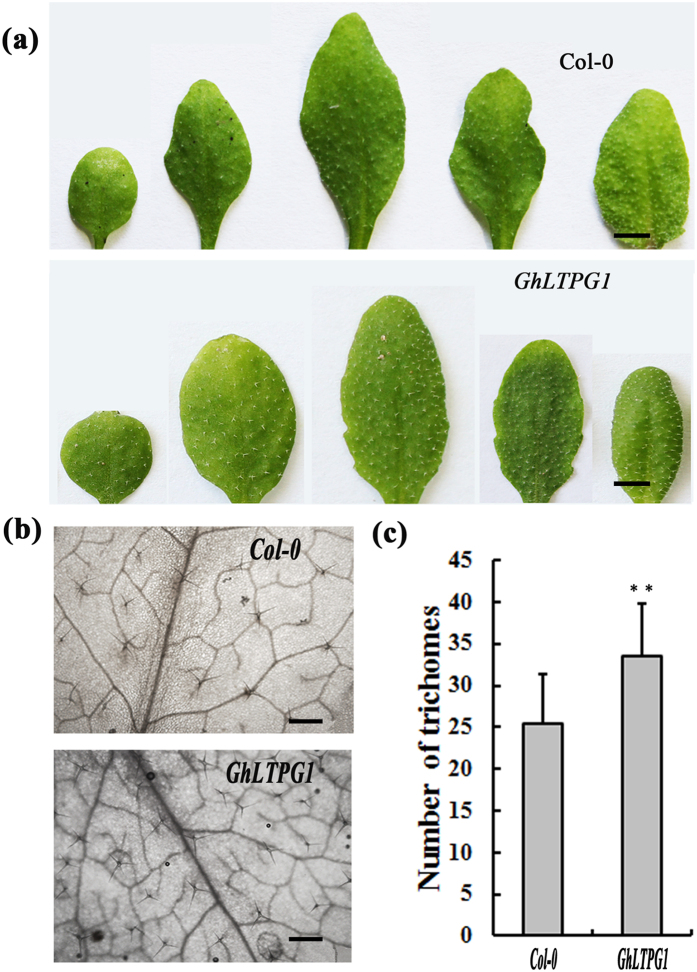
Ectopic expression of *GhLTPG1* in *Arabidopsis* results in the increased leaf trichomes. (**a**) Increased numbers of trichomes are observed on rosette leaves of *GhLTPG1*-expressing plants (*GhLTPG1*) than wild-type plants (Col-0). Bars = 5 mm. (**b**) The transgenic *GhLTPG1* plants (*GhLTPG1*) have more trichomes than wild-type plants in magnified leaf area. Bars = 500 μm. (**c**) The statistical number of leaf trichomes in transgenic *GhLTPG1* is higher than that of WT plants. Double asterisks represent the significant difference between transgenic *GhLTPG1* plants and wild-type (P < 0.01) in Student’s t-test.

Due to the similarities in the transcription factors and the mechanisms controlling arabidopsis leaf trichome and cotton fiber development, Arabidopsis is a good system in which to dissect gene function^36^,^37^. To gain further insight into the function of *GhLTPG1*, *GhLTPG1* was ectopically expressed in *Arabidopsis* and observation of the transgenic lines revealed the denser leaf trichomes (Fig. 8a,b). The trichome number of *GhLTPG1*-expressing plants was 32 ± 0.8/cm^2^, approximately 20% more than that of wild type (25 ± 1.0/cm^2^) (Fig. 8c). Analysis of the transcriptional level of genes including *MYB2L*, *GL2, MYB23, TTG2* and *TT8*, which involve in trichome development and epidermal cell fate determination during the trichome development^36^,^37^,^38^,^39^, revealed the significant increase (1.5 to ~2-fold) of these genes in *GhLTPG1*-expressingplants (Fig. S5), indicating that *GhLTPG1* enhances the trichome numbers by stimulating the expression of epidermal cell fate-determining genes.

## Discussion

Phosphatidylinositols serve as the constituents of fiber cell membrane and important signaling molecules during cotton fiber elongation^6^,^11^. The notable increase of PIs in cotton fiber occurs at the early stage of fiber elongation^6^,^30^, reflecting a large requirements for the assembly of expanding cell membranes and the membrane maintenance in quick developing fibers. In Arabidopsis, decreased *AtLTPG1* resulted in less wax loaded in the stem surface^26^. AtLTPG4 insertion mutant exhibited irregular seed coat and less fatty acid embedded^25^. PI is the second most abundant polar lipid, averaging 18% by weight of the total polar lipids in elongating fibers^6^. It is not clear which protein involved in PIs transport into fiber cell membrane. The present study indicates that GhLTPG1 binds to phosphatidylinositol monophosphates and delivers them to the fiber cell surface. Based on the results of *in vitro* and *in vivo* binding and transportation, as well as the phenotypes of GhLTPG1 VIGS plants, it is proposed that PIs, transported by GhLTPG1, affects the fiber length.

### PIs is transported by GhLTPG1 from the outer integument of ovules to fiber initials

Many *LTP* gene families are highly expressed during cotton fiber development^8^,^9^. Among the detected LTP gene families, *GhLTPG1* gene is only one that is richly expressed in elongating fibers^27^. *GhLTPG1* gene expression is restricted to the outer integuments of cotton ovules and fibers (Fig. 1c), suggesting that the outer integuments are the sources of PI for the quick growth of fiber cells. Furthermore, GhLTPG1 protein only localizes in the outer integument of seedcoat, the site of fiber initiation, supporting that GhLTPG1 transports the PIs from the outer integument of cotton ovule into fiber cell. The colocalization of GhLTPG1 protein with PtdIns3P or PtdIns4P demonstrated that GhLTPG1-PtdIns3P or GhLTPG1-PtdIns4P protein complex moves in small vesicles and targets to plasma membrane (Fig. 5, Video S1). Based on these results, it is hypothesized that PI molecules transported by GhLTPG1 is firstly from the outer integument to fiber initials, and then targets to the plasma membrane.

### PtdInsPs transported by GhLTPG1 affects cotton fiber elongation

LTPs in yeast transport PA or PE from the synthesis site to the developing organelles^40^,^41^. However, little evidence shows that LTPG or LTP protein in cotton acts as the PtdInsPs transporter during fiber elongation^42^,^43^. Unlike the *AtLTPG1* functions in stem cuticle deposition^18^,^19^,^26^, *GhLTPG1* gene is not expressed in stem, and GhLTPG1 protein specifically binds to and transports PtdInsPs. The distinctive expression pattern, polar lipids binding and transporting ability of GhLPTG1 help to further explore the functions of both GhLTPG1 and PtdInsPs in fiber elongation.

Reduced expression of *GhLTPG1* gene by VIGS resulted in reduced contents of PtdInsPs in cotton fibers (Fig. 7). Recent comparative lipidomics analysis of ovules between Xu142 and its fiberless mutant indicated that the total amounts of PA, PS, PC, and PE are similar in ovules at 10 DPA and exogenously added PtdInsPs in ovule culture significantly promoted fiber growth, while no differences were observed when PA, PC, PE, PG, and PS were added to the medium^30^. These results further suggest the importance of PIs for fiber elongation, and that GhLTPG1 participates in the transport of polar lipids to the fiber cell membrane.

The PI content in both wild type and *GhLTPG1* VIGS plants increased continuously during development of fiber cells, while PI increase in *GhLTPG1* VIGS plants is significantly slower than wild type at early stage of fiber development (0–6 DPA), which is consistent with the suppressed *GhLTPG1* expression and decreased fiber length from 0 to 6 DPA. Further, repressed the expressions of *GhLTPG1* reduced PI content and the lower expressions of fiber-elongation genes like *ACTIN1, EXPANSIN 1* and *CESA* genes in VIGS plants. Thus, it is proposed that PI molecules directly participate in cell expansion and cellulose synthesis, and affect fiber elongation velocity and mature fiber length. Interestingly, the differences in PI content between wild type and VIGS cotton plants are not obvious at the later stages of fiber elongation (after 6 DPA), suggesting that fiber cells may not obtain PIs from cotton ovules further, or there are other LTPG proteins responsible for the transport of polar lipids after 6 DPA.

The trichome development of *Arabidopsis* shares similar transcriptional regulatory network or module (MYB-bHLH-WD40) with cotton fiber^36^,^37^. Expression of *GhLTPG1* in Arabidopsis results in significantly increased leaf trichomes, which is in well accordance with the trichome surface localization of GhLTPG1 and the enhanced expression levels of trichome-regulating genes.

In conclusion, we demonstrated the specific expression and function of *GhLTPG1* in elongating cotton fibers, as well as the role of GhLTPG1 in phospholipids transport.

## Materials and Methods

### Plant materials and growth condition

Cotton cultivars (*Gossypium hirsutum* L.) Coker312, Xu142, and the fiberless mutant Xu142*fl* were used in this study. The plants were grown in the experimental field at Shanghai Jiao Tong University, Shanghai, China. When plants had grown for 90 days, ovules and fibers at different stages were removed carefully from developing bolls. Roots, stems, and leaves were collected from 15-day-old seedlings for gene expression analysis.

### *GhLTPG1* gene cloning and expression pattern analysis

Based on the results of the RNA-seq analysis, a putative *LTPG* gene was found being up-regulated during early stages of fiber elongation. The relevant *LTPG* gene was amplified by PCR using cDNAs synthesized from mRNA isolated from elongating fibers of Coker312 as the template. The amplified products were cloned into the *pMD19-T* vector (TaKaRa, Japan) and confirmed by DNA sequencing. Expression pattern of *GhLTPG1* was analyzed by Real-time quantitative PCR (qRT-PCR) and primers are listed in Supplementary Table S1.

qRT-PCR analysis was performed in a DNA Engine Option 3 System (MJ Research, USA) using SYBR Premix Ex-Taq (Takara, Japan). Each sample was repeated at least three times. Transcriptional changes were calculated based on the comparative ΔCT method^44^.

The deduced amino acid sequence of cotton GhLTPG1 was aligned with the *Arabidopsis* LTPGs using BioEdit and ClustalX. Predicted molecular weight, iso-electric point, functional domains, and amino acid signal peptides were calculated using the ExPASy online servers (http://cn.expasy.org/tools).

### Subcellular localization studies of GhLTPG1 and the truncated proteins

To investigate the subcellular localization of GhLTPG1 protein and the effect of various domains on protein localization, coding region of *GhLTPG1* and the truncated fragments encoding different domains (GhLTPG1ΔSP/67–588 bp, GhLTPG1ΔGPI/1–492 bp, and GhLTPG1ΔSPΔGPI/67–492 bp) were cloned into the *pEarlyGate101* vector to generate *pEarlyGate 101*–*35* *S::GhLTPGs-eYFP::NOS* constructs. The plasmids were then transformed into *Agrobacterium tumefaciens* strain GV3101 according to description^44^. Three week-old tobacco leaves were then infiltrated with the *Agrobacterium* strain. Protein subcellular localization was detected two days after inoculation by visualizing the fluorescence using laser confocal microscopy (Leica TCS SP5).

### *In situ* RNA hybridization analysis

Tissue fixation and embedding, *in situ* hybridization, and signal detection were performed according to previous description^45^. To prepare RNA probes, a 588-bp fragment was amplified by PCR using RNA *in situ* probe primers (Supplementary Table S1). The fragment was then cloned into the *pGEM-T-*Easy vector (Promega, USA) and used as the template for *in vitro* transcription. Digoxigenin-labeled sense or antisense RNA probes were prepared using a Digoxigenin SP6/T7 Labeling Kit (Roche, Germany). The hybridization slides were observed and photographed under an Olympus BX51 microscope (Olympus, Japan).

### Tissue expression pattern analysis of *GhLTPG1* in *Arabidopsis*

Tissue expression pattern of *GhLTPG1* was analyzed by qRT-PCR method as described above^44^. For promoter-GUS fusion analysis, the *GhLTPG1* promoter was cloned into *pCAMBIA1305.1* to drive the *GUS* gene expression. The resultant construct was transformed into *Arabidopsis thaliana* Col-0 using the *Agrobacterium*-mediated floral dipping method^46^. Different tissues including root, stem, flower, and seeds of T_1_ plants were treated with GUS staining solution and observed using an Olympus BX51 microscope (Olympus, Japan)^23^,^44^.

### Ectopic expression of *GhLTPG1* and the variants in *Arabidopsis*

To analyze the function of *GhLTPG1*, the *GhLTPG1* and truncated regions deleting different domains (GhLTPG1ΔSP/67–588 bp, GhLTPG1ΔGPI/1–492 bp and GhLTPG1ΔSPΔGPI/67–492 bp) were cloned into the *pEarlyGate101* vector to generate the expression cassettes *pEarlyGate101*–*35* *S::GhLTPG1s-eYFP::NOS*. The constructs were transformed into *A. tumefaciens* GV3101, and then introduced into *Arabidopsis* Col-0 plants using floral dip method^46^. The transgenic *GhLTPG1* plants were self-crossed to generated T_3_ homozygous lines. Three T_3_ lines were randomly selected to analysis their trichome phenotypes. Fifteen transgenic plants from each independent T_3_ line and 10 wild-type plants were grown under same conditions. Leaf trichome, and the number of trichomes of mature leaves were observed or calculated at seedling stages (5–10 rosette leaves appeared).

### GhLTPG1 *in vitro* lipid binding assays

The nucleotide sequence encoding the LTP domain of GhLTPG1 was cloned into the *pGEX4T-3* vector (Novagen, Germany), and confirmed by DNA sequencing. The plasmids were transformed into *E. coli BL21*(DE3) cells. The positive clones were first grown at 37 °C to an OD_600_ of 0.60, and IPTG was then added to a final concentration of 1 mM to induce GhLTPG1 protein expression. After 4 hours, the cells were collected by centrifugation and re-suspended. The cell suspension was lysed by sonication and then centrifugated at 12,000 rpm for 30 min at 4 °C. The GhLTPG1 proteins were purified from the resulting supernatants on a GST-binding column.

The PIPs (phosphatidylinositol phosphates) and Membrane Lipid strips (Echelon Biosciences, USA) were first blocked in PBS-T (PBS plus 0.1% Tween-20) with 3% BSA for 1 h at room temperature. After discarding the blocking buffer, 25 μg GhLTPG1 protein was added in 5 mL blocking buffer and incubated with the membrane for two hours at room temperature. Membranes were washed with PBS-T for 3 times (10 min for each time). Anti-GST monoclonal antibody (Novagen, Germany) diluted 1:10,000 in blocking buffer was added and incubated at 4 °C overnight. The membranes were washed three times with PBS-T, then added with anti-mouse antibody (Promega, USA, 1:7,500 dilution) and incubated for 1 hour at room temperature. After washing with PBS-T three times, the protein was detected with 1 mL of the BCIP/NBT detection system (TIANGEN, China).

### Phospholipid transfer assay in plants

Colocalization analysis was performed to determine whether GhLTPG1 possesses PtdInsP transport activity^47^. The coding region (without the termination codon) of the full-length GhLTPG1 protein or the LTP domain was cloned into *pEarleyGate101* to generate GhLTPG1-eYFP and LTP-eYFP fusion proteins. These constructs and different PtdInsP marker plasmids (mCherry-fused protein) were transformed into *A. tumefaciens* GV3101^44^. For infiltration, equal volumes of suspensions of *Agrobacterium* strains carrying different constructs were mixed prior to infiltration. The resuspended cells were infiltrated into leaves of tobacco plants as described previously. The tobacco cells were observed under a confocal microscope two days after infiltration.

### Phospholipid transfer assay between liposomes

Liposomes preparation and lipids transfer activity assay were performed according to the modified method reported by Connerth *et al.*^41^. PtdInsPs (PtdIns3P, PtdIns4P, PtdIns5P and PtdIns-4,5-P_2_) and PC were purchased from Echelon Biosciences (USA). We prepared the acceptor liposomes by 600 nmol PC alone, and the acceptor liposomes by PC mixed with PtdInsPs [PtdIns3P, PtdIns4P, PtdIns5P and PtdIns(4,5)P_2_] in the molecular ratio of 9:1 (donor) in chloroform and dried under nitrogen. Acceptor lipids were then rehydrated in 1 mL rehydration buffer (500 mM Tris-HCl, 100 mM NaCl, and 1 mM DTT, pH 7.5) and donor lipids were rehydrated in rehydration buffer containing 25% sucrose. The rehydrated samples were subjected into 6 cycles of freezing and thawing at dry ice and 42 °C water bath. The lipid samples were then extruded by a liposome extruder through a polycarbonate membrane (0.1 μm filters for acceptor liposomes, 0.4 μm filters for donor liposomes) to produce an optically clear suspension of small unilamellar liposomes according to the manufacturer’s instructions (Avanti, USA). Liposomes were stored on ice and used within two days.

The donor liposomes were pelleted at 16,000 g for 30 min and washed with the buffer (10 mM HEPES, 250 mM NaCl, pH 7.5). Recombinant GhLTPG1 protein (1.0 μM) was incubated with the donor and acceptor liposomes (each corresponding to 2.0 mM total lipids) in 400 μL assay buffer at room temperature for 1 hour. After centrifugation at 16,000 g for 30 min, chloroform and methanol were added into the supernatants in a ratio of 1.25:2.5:1 (v/v/v). After vortexing well, chloroform and H_2_O (1.25 times of the supernatants volume each) were then added and vortex gently and thoroughly. The lipids film was generated by evaporating the organic phase separated by centrifuging the mixture at 1000 RPM for 5 min.

Transferred lipids were dissolved in chloroform/methanol (1:2) and analyzed by flow injection using a Shimadzu CBM-20 A Lite HPLC system (Shimadzu, Japan) with a solvent mixture of 10 mM ammonium acetate in methanol. A flow gradient was performed starting with 100 μl/min for 5 sec followed by 1.7 min of 30 μl/min and 150 μl/min for 1 min. Mass spectrometric analysis was done on a 5500 QTrap triple quadrupole mass spectrometer (AB Sciex, USA) equipped with a Turbo V electrospray ion source under following settings: source temperature, 300 °C; curtain gas, 25; collision gas, medium; two ion source gases, 30; ion spray voltage, 4500 V for PtdInsPs and 5500 V for PI; entrance potential, 10 V. Scan rate was 200 Da/s. PtdInsPs measurement was performed in negative ion mode by perform multiple reaction monitoring (MRM) scans for 889.4/321 Da and 442.2/241 Da at CE of −55 eV and −30 eV, respectively. PtdInsP_2_ (phosphatidylinositol bisphosphate) measurement was performed in negative ion mode by MRM scans for 969.4/401 Da and 484.2/321 Da at CE of −55 eV and −32 eV, respectively. PI measurement was performed in positive ion mode by MRM scan for 828.6/551 Da at CE of 30 eV.

### Observation of fiber development in cotton *GhLTPG1* Virus induce gene silencing plants

The *GhLTPG1* specific region was cloned into the pTRV2 plasmid and confirmed by sequencing. pTRV1, pTRV2, and pTRV2 derivatives were introduced into *A. tumefaciens* strain LBA4404^48^,^49^. *Agrobacterium* cells carrying pTRV1 and pTRV2 or the pTRV2 derivatives were mixed in a 1:1 ratio and then used in vacuum infiltration. Whole cotton plants (2 weeks old) were submerged in the *Agrobacterium* cell suspension and subjected to a vacuum of 80–100 kPa for 1 min to allow the *Agrobacterium* to enter the plant tissues^48^,^49^. After infiltration, cotton plants were transferred into pots. All virus-induced-gene-silencing (VIGS) experiments were repeated at least three times with >40 plants for each construct.

Four weeks after infiltration, total RNA was extracted from leaves of the infiltrated plants. qRT-PCR was used to confirm that the PTRV virus had successfully suppressed *GhLTPG1* expression in the VIGS plants. Cotton ovules at different developmental stages (0, 3, 6, and 9 DPA and the mature fiber) from VIGS-positive plants and control plants (pTRV1 and pTRV2) were collected for gene expression analysis and fiber length measurement.

The expressions of genes (*GhCESA1, GhPEL, GhACT1, GhEX1, GhPFN2, GhFLA1, GhFLA2*) related to fiber elongation in cotton ovules were analyzed by qRT-PCR. Results were normalized using ubiquitin gene expression as the internal control, and repeated at least three times. Primers are listed in the Supplementary Table S1.

### Lipid content analysis of cotton ovules during fiber elongation

Cotton ovules (at 0, 3 DPA) and fibers (at 6, and 9 DPA) from VIGS plants and control plants were collected for lipid content analysis. All collected materials were immediately dipped into 2 mL preheated isopropanol (75 °C) and total lipid extraction was performed according to the method described by Wanjie^6^. Various PtdInsP molecules (Sigma-Aldrich, USA) were added to the extracts as standards to check the content of different lipids. Lipids in the samples were identified by comparison with standards and the amount of each polar lipid was calculated based on the amount of the internal standard.

## Additional Information

**How to cite this article**: Deng, T. *et al.* GhLTPG1, a cotton GPI-anchored lipid transfer protein, regulates the transport of phosphatidylinositol monophosphates and cotton fiber elongation. *Sci. Rep.* **6**, 26829; doi: 10.1038/srep26829 (2016).

## Supplementary Material

Supplementary Information

Supplementary Video 1
