# Supplementary material for: GhLTPG1, a cotton GPI-anchored lipid transfer protein, regulates the transport of phosphatidylinositol monophosphates and cotton fiber elongation
Source: Sci Rep. 2016 Jun 17;6:26829. doi: 10.1038/srep26829 (PMC4911556; doi:10.1038/srep26829)
Supplement: Supplementary Information [file srep26829-s1.pdf]

Supporting information

GhLTPG1, a cotton GPI-anchored lipid transfer protein, regulates the transport of phosphatidylinositol monophosphates and cotton fiber elongation

Ting Deng[1§], Hongyan Yao[2§], Jin Wang[3§], Jun Wang[1], Hongwei Xue[2,*], Kaijing Zuo[1,*]

[1]Plant Biotechnology Research Center, School of Agriculture and Life Sciences, Shanghai Jiao Tong University, Shanghai 200240, China

[2] National Key Laboratory of Plant Molecular Genetics, Shanghai Institute of Plant Physiology& Ecology, Chinese Academy of Sciences, 300 Fenglin Road, 200032 Shanghai, China

[3] Biotechnology Research Institute, Chinese Academy of Agricultural Sciences, Beijing, 100081, China

§ These authors contribute equally to this paper.

* Correspondence to Kaijing Zuo, kjzuo@sjtu.edu.cn or to Hongwei Xue, hwxue@sibs.ac.cn.

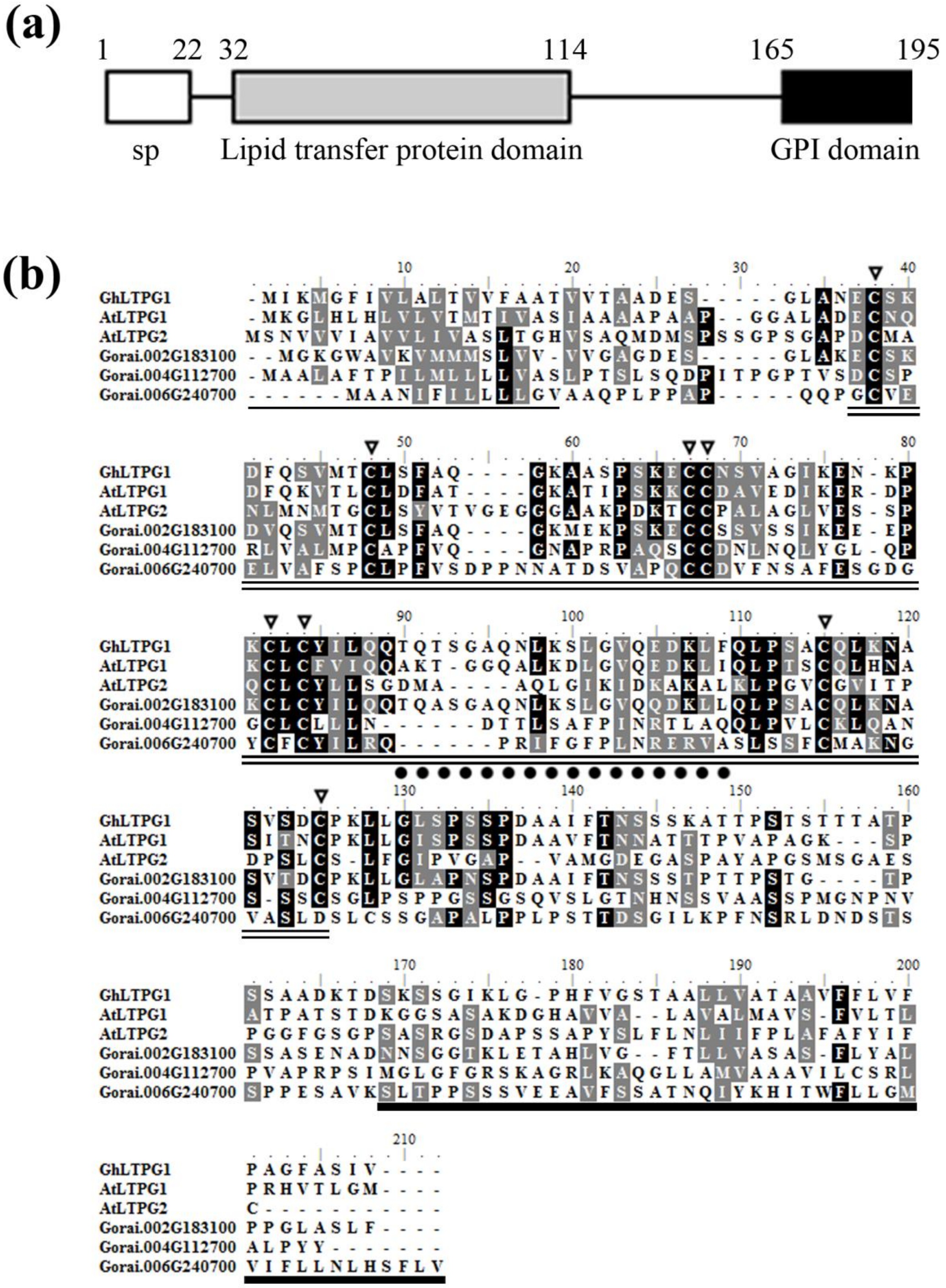

Fig.S1 GhLTPG1 has typical structure of lipid transporter protein, and shows limited similarity with LTPG proteins from cotton and *Arabidopsis.*

(a) Schematic representation of the domain structure of GhLTPG1. SP, signal peptide; GPI-domain, Glycosylphosphatidylinositol-anchored domain.

(b) Sequence alignment of GhLTPG1 and two LTPG homologs from Arabidopsis.

The single underline and double underline indicate the signal peptide domain and the lipid transport domain respectively. GPI-domain at C-terminal is shown by the black line. The conserved Cys residues are indicated by inverted triangles. The dotted line represents the unconserved region of LTP domain between GhLTPG1 and the three most closely-related LTPGs. The sequence used in virus induced gene silencing locates from 90th to 201th amino acid in GhLTPG1.

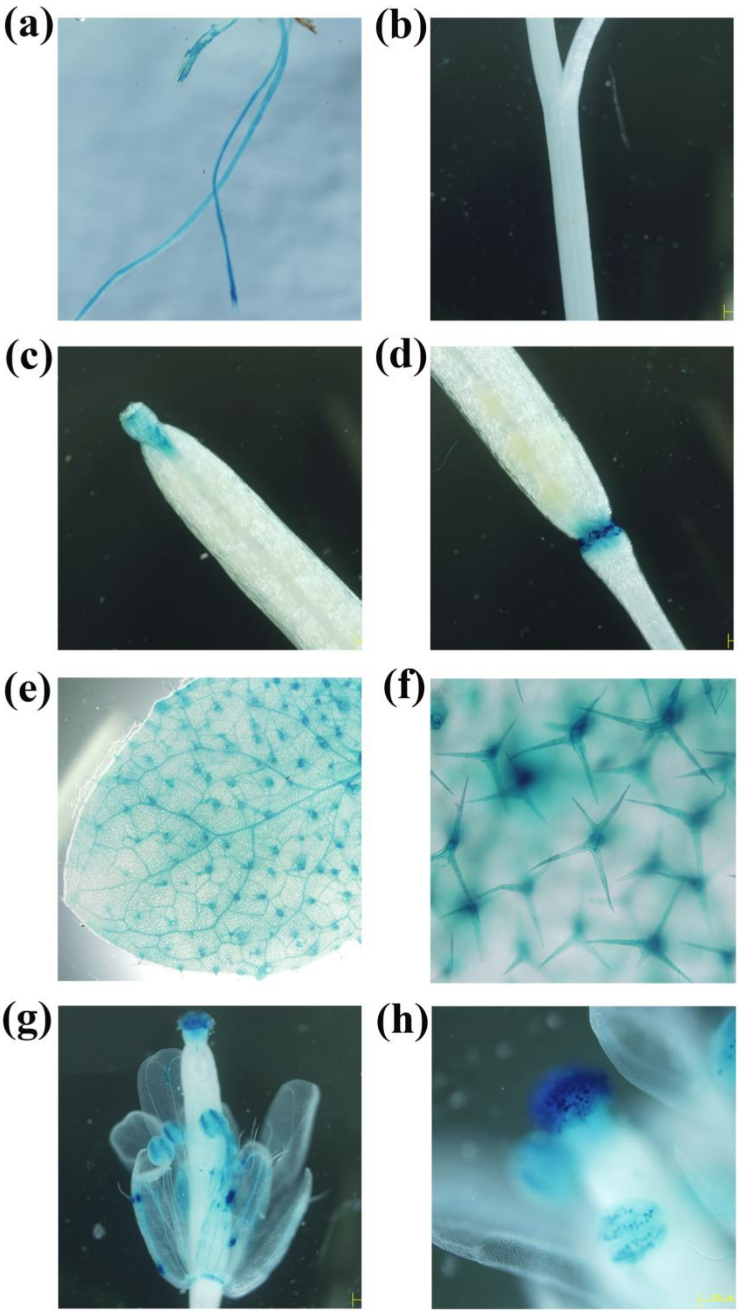

Fig.S2 *GhLTPG1* gene promoter activity is restricted to the *Arabidopsis* root, trichomes in different tissues.

(a) *GhLTPG1* gene strongly expresses in root.

(b) *ProGhLTPG1::GUS* expression is absent from stem.

(c-d) The signals of *GhLTPG1* gene are detected at the top and bottom of siliques.

(e-f) *ProGhLTPG1::GUS* activity is detected on the trichomes of leaf.

(g-h) *ProGhLTPG1::GUS* is expressed in anther and stigma, and trichomes on petals.

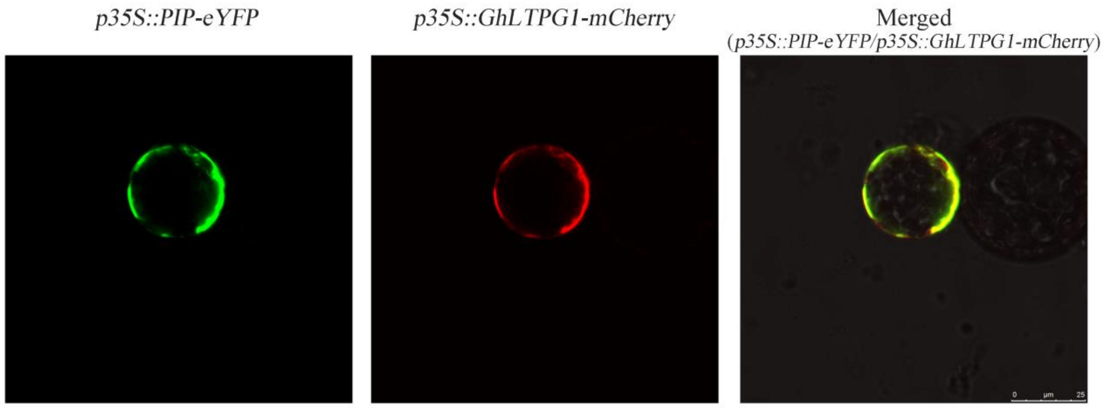

Fig.S3 *GhLTPG1* colocalizes with AtPIP (membrane protein) on cell membrane.

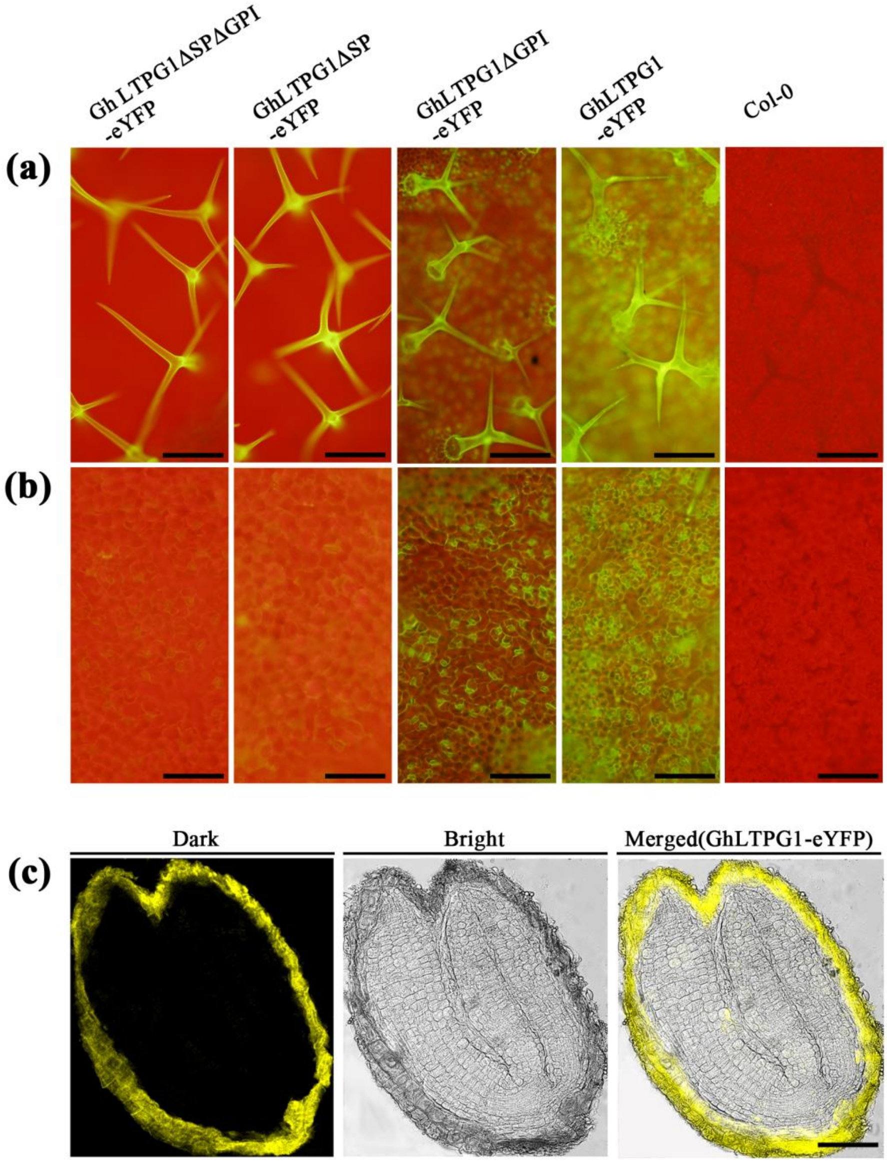

Fig.S4 GhLTPG1-eYFP protein localization in different tissues when expressed in *Arabidopsis*.

(a) GhLTPG1 and its truncated proteins fused with eYFP are localized in *Arabidopsis* trichome surface. Bars= 100 μm.

(b) GhLTPG1 truncated variants are unevenly localized in cell membrane of *Arabidopsis* leaf epidermis cells. Bars= 50 μm.

(c) GhLTPG1-eYFP is localized in outer integument of *Arabidopsis* seed coat.

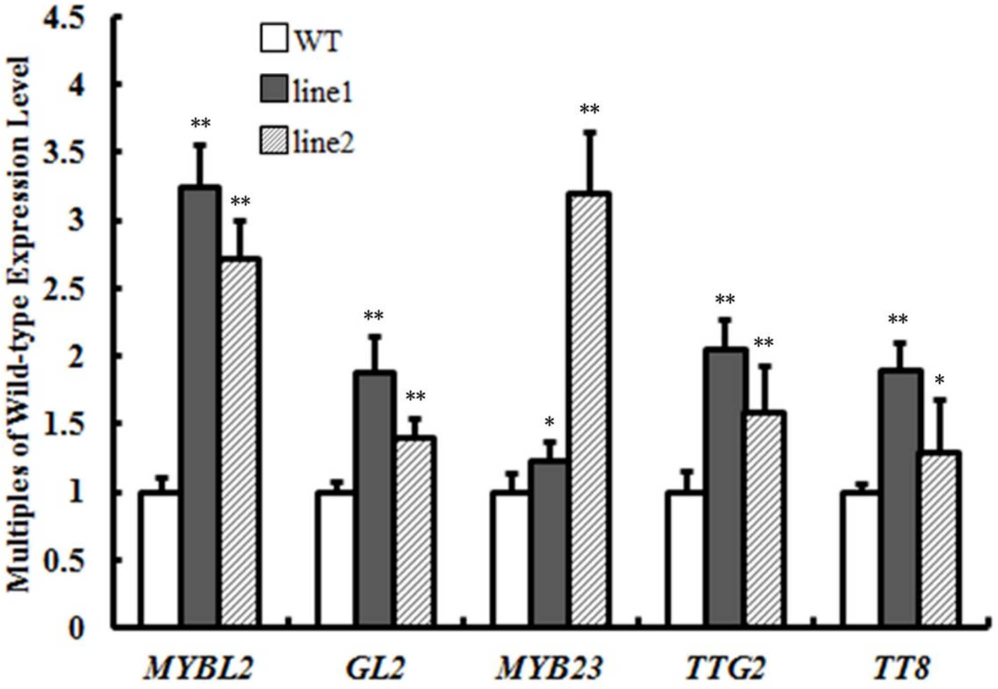

Fig. S5 Overexpressed *GhLTPG1* gene in *Arabidopsis* activates the expressions of trichome development-associated genes.

The gene expression levels are shown in fold changes in transgenic *GhLTPG1* lines compared with wild type (*, P <0.05; **, P <0.01). WT, Col-0; line 1 and line 2, two independent *GhLTPG1* overexpressed lines. *MYBL2*, AT1G71030; *GL2*, AT1G79840; *MYB23*, AT5G40330; *TTG2*, At2g37260; *TT8*, AT4G09820.

Video S1. The video, showing that GhLTPG1-eYFP-PtdIns3P biomarker (2xFYVE^{mHrs}-mCherry) **protein complexes in the cytoplasm move in vesicles along the filaments track.**

GhLTPG1-eYFP and PtdIns3P biomarker were observed separately, and the overlay shows the movement of GhLTPG1-PtdIns3P complexes. Arrows and asterisks indicate the complexes move along the filament from plasma membrane to plasma

membrane and from cytoplasm to plasma membrane respectively. It took about 7.2 seconds to capture the yellow and red fluorescence signals separately for one Frame, about 158 seconds were used for 22 frames shown in the video, so the 22 seconds video shows 158 seconds of movement in the cytoplasm.

Supplementary table 1 Primers used in this paper.

Vector and genes		primer sequence (5’-3’)	
GhLTPG1 gene cloning		Forward	AAAAAAATAGAGCAGTGAAGAAAG
		Reverse	GGAAAAATAAACGAAAATCATCATAAT
GhLTPG1 promoter::GUS construct		Forward	ACGCGTCGACCAGTTTGCCGCCTGCCGTTC
		Reverse	CTCATGCCATGGCCCCTAAACTATTGAAGCAAATC
GhLTPG1:eYFP construct	LTP-eYFP	Forward	ATGGAGAGTGGGTTAGCGAATGAGTG
		Reverse	AAGCTTGATTCCACTGGATTTG
	LTP-GPI-eYFP	Forward	ATGGAGAGTGGGTTAGCGAATGAGTG
		Reverse	AACTATTGAAGCAAATCCAGCTG
	SP-LTP-eYFP	Forward	ATGATCAAAATGGGTTTTATTGTTT
		Reverse	AAGCTTGATTCCACTGGATTTG
	SP-LTP-GPI-eYFP	Forward	ATGATCAAAATGGGTTTTATTGTTT
		Reverse	AACTATTGAAGCAAATCCAGCTG
GhLTPG1 RNAi vector		Forward	GAGAGTGGGTTAGCGAATGAGTG
		Reverse	AAGCTTGATTCCACTGGATTTG
RNA in situ probe		Forward	AGCAGTGAAGAAAGATGATC
		Reverse	CAACAATAAATAAACCTCCAAATC
GhLTPG1:GST construct		Forward	ATGGAGAGTGGGTTAGCGAATGAGTG
		Reverse	CTAAAGCTTGATTCCACTGGATTTG
qRT-PCR		Forward	GAAGACGAAGAACAAGGGGAAG
		Reverse	AGCTCGGATACGATTGATAACG
Poly-ubiquitin		Forward	CAAGCCGATGGTAACTGAAA
		Reverse	GATTTGCTATCGGTTTTATC
GhCESA1		Forward	CAGTGGATTACCCAGTGGACAA
		Reverse	GGCACCCACTTTCTTAGCAAAC
GhPEL		Forward	CTATGGTTGGAGGACGATAT
		Reverse	TGTTCTTGTCTTGAGTATAGGA
GhACT1		Forward	TAACCGATGCCTTGATGA
		Reverse	AATAGTAATAACTTGTCCGTCAG
GhEX1		Forward	TTGTCCACCTAACTATGCT
		Reverse	GCTTCGTTATCAACACCAT
GhPFN2		Forward	GGAATCTATGATGAACCAATGA
		Reverse	TAATACGGAGACGCTTCG
GhFLA1		Forward	AAGTTCCCTCTCAATGTCA
		Reverse	GTGGTAGCCTTCTTATTCTTC
GhFLA2		Forward	ACACTGACCAAGGCATTA
		Reverse	CGGAATTGTCTGTGAAGTT
